# Characterization of eleven monosomic alien addition lines added from *Gossypium anomalum* to *Gossypium hirsutum* using improved GISH and SSR markers

**DOI:** 10.1186/s12870-016-0913-2

**Published:** 2016-10-07

**Authors:** Xiaoxiao Wang, Yingying Wang, Chen Wang, Yu Chen, Yu Chen, Shouli Feng, Ting Zhao, Baoliang Zhou

**Affiliations:** 1 State Key Laboratory of Crop Genetics & Germplasm Enhancement, Nanjing Agricultural University, Nanjing, 210095 China; 2 Key Laboratory of Cotton Breeding and Cultivation in Huang-Huai-Hai Plain, Ministry of Agriculture, Cotton Research Center of Shandong Academy of Agricultural Sciences, Jinan, 250100 Shandong People’s Republic of China

**Keywords:** *Gossypium hirsutum*, *Gossypium anomalum*, Chromosome, Monosomic alien addition line, Genomic in situ hybridization, Microsatellite marker

## Abstract

**Background:**

*Gossypium anomalum* (BB genome) possesses the desirable characteristics of drought tolerance, resistance to diseases and insect pests, and the potential for high quality fibers. However, it is difficult to transfer the genes associated with these desirable traits into cultivated cotton (*G. hirsutum*, AADD genome). Monosomic alien addition lines (MAALs) can be used as a bridge to transfer desired genes from wild species into *G. hirsutum*. In cotton, however, the high number and smaller size of the chromosomes has resulted in difficulties in discriminating chromosomes from wild species in cultivated cotton background, the development of cotton MAALs has lagged far behind many other crops. To date, no set of *G. hirsutum-G. anomalum* MAALs was reported. Here the amphiploid (AADDBB genome) derived from *G. hirsutum* × *G. anomalum* was used to generate a set of *G. hirsutum-G. anomalum* MAALs through a combination of consecutive backcrossing, genomic in situ hybridization (GISH), morphological survey and microsatellite marker identification.

**Results:**

We improved the GISH technique used in our previous research by using a mixture of two probes from *G. anomalum* and *G. herbaceum* (AA genome). The results indicate that a ratio of 4:3 (*G. anomalum* : *G. herbaceum*) is the most suitable for discrimination of chromosomes from *G. anomalum* and the At-subgenome of *G. hirsutum*. Using this improved GISH technique, 108 MAAL individuals were isolated. Next, 170 *G. hirsutum-* and *G. anomalum*-specific codominant markers were obtained and employed for characterization of these MAAL individuals. Finally, eleven out of 13 MAALs were identified. Unfortunately, we were unable to isolate Chrs. 1B^a^ and 5B^a^ due to their very low incidences in backcrossing generation, as these remained in a condition of multiple additions.

**Conclusions:**

The characterized lines can be employed as bridges for the transfer of desired genes from *G. anomalum* into *G. hirsutum*, as well as for gene assignment, isolation of chromosome-specific probes, development of chromosome-specific “paints” for fluorochrome-labeled DNA fragments, physical mapping, and selective isolation and mapping of cDNAs/genes for a particular *G. anomalum* chromosome.

**Electronic supplementary material:**

The online version of this article (doi:10.1186/s12870-016-0913-2) contains supplementary material, which is available to authorized users.

## Background

Cotton is the leading natural textile fiber crop in the world. Approximately 5 % of the world’s arable land is used for cotton planting, generating about $630.6 billion in 2011 [1]. Cotton belongs to the *Gossypium* genus of Malvaceae, which contains five tetraploid species (2n = 4× = 52, AADD genome) and approximately 45 diploid species (eight genomes from A to G and K, 2n = 2× = 26) [2]. Upland cotton (*G. hirsutum*) is the most widely cultivated species and its production accounts for over 95% of the world’s cotton production [3]. During the development of its cultivars, cotton has been subjected to long-term artificial selection, which narrowed its genetic base and gave rise to several difficulties in breeding. Cotton breeders face a scarcity of genetically diverse resources, therefore expanding the genetic base of cotton cultivars is imperative. Wild or untapped species have many excellent characteristics and contain abundant desirable genes, which have yet to be unlocked by pre-breeding. *G. anomalum* (2n = 2× = 26, BB genome) which is native to Africa, mainly Angola and Namibia [2], has the favorable characteristics of drought tolerance and resistance to diseases (cotton wilt, angular leaf spot) and insect pests (springtails, aphids): more importantly, it also possesses genes with the potential to produce high quality fibers (good fiber strength and fineness) [4] and cytoplasmic male sterility [5–7]. However, it is difficult to transfer these desirable genes into cultivated cotton through conventional breeding methods due to the isolation of wild species from cultivated species, which limits chromosome pairing and genetic recombination.

Monosomic alien addition lines (MAALs) contain only one alien chromosome in addition to the receptor background chromosomes. MAALs can be used as a bridge to transfer desired genes from wild species into *G. hirsutum* [8]. Over the past two decades, MAALs have been widely available for numerous crops [9], and these can be used for effectively identifying favorable genes in wild species, allowing for more accurate and faster transfer of such genes to create introgression lines, the effect of specific alien chromosomes to be examined, homeologies with chromosomes of cultivated species to be compared [10, 11], and physical maps of specific chromosomes to be constructed [12]. In cotton, however, the high number and smaller size of the chromosomes has resulted in difficulties in discriminating chromosomes from wild species in cultivated cotton background, therefore the development of cotton MAALs has lagged far behind many other crops. No set of cotton MAALs was reported until cotton molecular genetic maps were constructed and a genomic in situ hybridization (GISH) technique for cotton was developed. Previously, only one complete set of *G. hirsutum-G. australe* MAALs had been developed using simple sequence repeat (SSR) markers and GISH [9, 13, 14]. Two *G. hirsutum-G. somalense* MAALs and several *G. hirsutum-G. sturtianum* MAALs have also been obtained [11, 15].

In this study, the *G. hirsutum-G. anomalum* hexaploid was used as a maternal parent in the continuous backcrossing with upland cotton (recipient parent, *G. hirsutum* acc. TM-1), and eleven MAALs were isolated using GISH and SSR markers. These MAALs may be useful for mining and transferring favorable genes from *G. anomalum* into *G. hirsutum* on a genome-wide scale, mapping genes on chromosomes, analyzing genome structure and evolution, and micro-cloning for chromosome-specific library construction.

## Results

### Alien chromosomes from *G. anomalum* in *G. hirsutum* were examined by the improved GISH

**Fig. 1:**
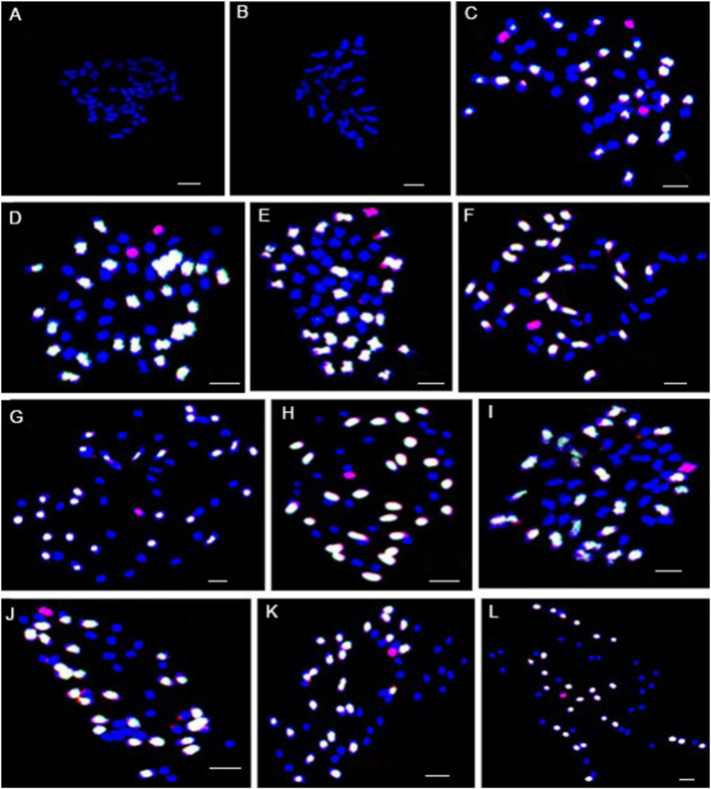
Genomic in situ hybridization of the putative alien chromosomes of *G. anomalum* in the *G. hirsutum* background using two *G. herbaceum* and *G. anomalum* probes. Genomic DNA from *G. anomalum* and *G. herbaceum* was labeled with digoxigenin-11-dUTP and Bio-16-dUTP by nick translation, respectively. Chromosomes of the At-subgenome of *G. hirsutum* were cross-hybridized with both the *G. anomalum* and *G. herbaceum* probes and produced white signals and chromosomes of the Dt-subgenome of *G. hirsutum* were stained with 4′,6-diamidino-2-phenylindole (DAPI) and produced blue signals. Chromosomes from *G. anomalum* were hybridized with *G. anomalum* probe and produced red signals. **a** mitotic chromosome spread of the 52 chromosomes of *G. hirsutum*. **b** mitotic chromosome spread of the 26 chromosomes of *G. anomalum*. **c**–**l** mitotic chromosome spread showing the 52 *G. hirsutum* (*white* and *blue*) chromosomes and three (**c**), two (**d**), and one (**e**, **f**, **g**, **h**, **i**, **j**, **k** and **l**) individual chromosomes of *G. anomalum* (*red*), respectively. Scale bar = 5μm

The GISH technique used in our previous research was improved as follows. Genomic DNA extracted from *G. anomalum* and *G. herbaceum* was labeled with digoxigenin-11-dUTP and Bio-16-dUTP (Roche Diagnostics, Mannheim, Germany) by nick translation, respectively. The labeled DNA was mixed at a variety of ratios for GISH analysis using chromosomes from the mitotic metaphases as target templates. The results indicate that a ratio of 4:3 is the most suitable for discrimination of chromosomes from *G. anomalum* and the At-subgenome of *G. hirsutum*. At this ratio the chromosomes from *G. anomalum* only hybridized with the *G. anomalum* probe to produce a red signal, while chromosomes of the At-subgenome of *G. hirsutum* cross-hybridized with both the *G. anomalum* and *G. herbaceum* probes to produce a white signal and chromosomes of the Dt-subgenome of *G. hirsutum* were stained with 4’,6-diamidino-2-phenylindole (DAPI) (Roche Diagnostics), producing a blue color. Therefore, the GISH technique has been improved and can be further used to differentiate chromosomes from *G. anomalum* and the At-subgenome of *G. hirsutum* (Fig. 1).

Progenies of the pentaploid of (*G. hirsutum* × *G. anomalum*) × *G. hirsutum* backcrossed with *G. hirsutum* were subjected to GISH to determine the number of alien chromosomes transferred from *G. anomalum* to *G. hirsutum* using visible fluorescent hybridization signals. Thirty eight individuals of the BC_1_ population were examined by GISH analysis (Additional file 1: Table S1). The analysis demonstrated that 27 (71.05 %) carried 2 to 6 alien chromosomes, and 6 (15.79 %) carried 7 to 9 alien chromosomes. Only two (5.26 %) individuals carried one chromosome, 6B^a^ and 13B^a^ of *G. anomalum*, resepctively. One (2.63 %) plant had no alien chromosomes and the final two (5.26 %) plants had 13 alien chromosomes from *G. anomalum* (Fig. 1; Table 1).

**Table 1:** Incidence of alien chromosomes in the BC_1_ to BC_2_ *G. hirsutum* × *G. anomalum* generations

Chromosome number	1B^a^	2B^a^	3B^a^	4B^a^	5B^a^	6B^a^	7B^a^	8B^a^	9B^a^	10B^a^	11B^a^	12B^a^	13B^a^	No. individuals
52	0	0	0	0	0	0	0	0	0	0	0	0	0	122
52 + 1	0	10	1	17	0	16	3	6	1	34	2	7	11	108
52 + 2	1	9	6	31	1	16	5	1	0	19	5	13	1	54
52 + 3	3	2	6	9	1	8	5	1	0	5	4	7	4	19
52 + 4	1	2	4	2	1	4	1	1	2	2	0	4	1	6
52 + 5	2	1	3	2	3	1	1	2	1	1	1	1	1	4
52 + 6	4	3	4	1	2	1	3	3	2	3	3	2	4	7
52 + 7	1	1	2	0	2	1	1	0	1	0	1	2	2	2
52 + 8	2	1	2	2	0	1	1	1	1	2	1	1	1	2
52 + 9	2	2	2	1	0	2	2	2	1	2	1	1	0	2
52 + 13	2	2	2	2	2	2	2	2	2	2	2	2	2	2
SUM	16	31	30	65	10	50	22	17	9	68	18	38	25	328
Incidence (%)	4.65	7.33	7.58	15.89	2.69	12.47	5.62	4.16	2.44	16.87	4.65	9.29	6.36	
Monosomic addition (%)	0.00	9.26	0.93	15.74	0.00	14.81	2.78	5.56	0.93	31.48	1.85	6.48	10.19	

A total of 290 individuals from the BC_2_ generation were further analyzed by GISH. The results indicated that 106 (36.55 %) individuals had one alien chromosome of *G. anomalum* and 121 (41.72 %) had no alien chromosomes in the *G. hirsutum* background. 50 (17.24 %) and 10 (3.45 %) individuals carried two and three alien chromosomes, respectively, and another 1 (0.34 %) carried four alien chromosomes. The results demonstrated that most of the BC_2_ individuals carried 0-1 alien chromosomes, and only a small number contained multiple alien chromosomes (Fig. 1; Table 1).

### Screening of a set of putative *G. anomalum* chromosome-specific SSR primer pairs

**Fig. 2:**
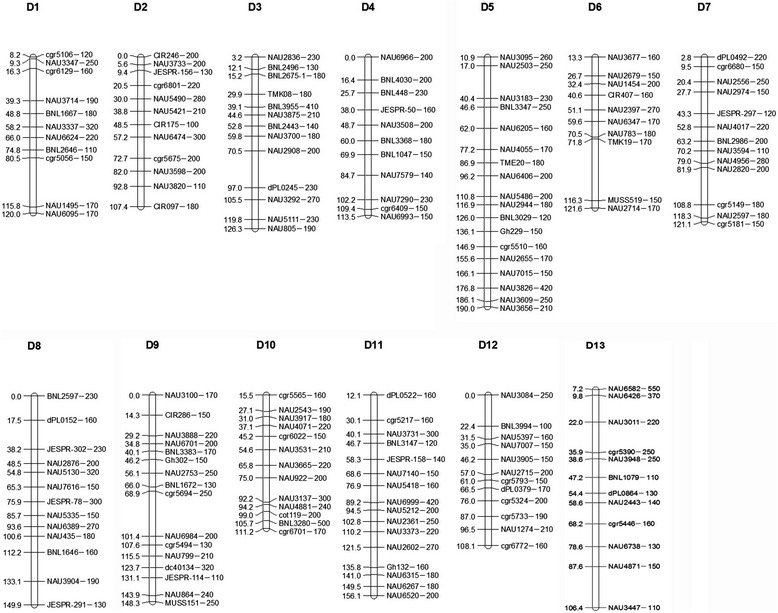
Genetic linkage map of *G. anomalum* chromosome-specific SSR markers based on the linkage map of tetraploid cotton reported by Zhao et al. (2012)

During the evolution of *Gossypium*, chromosomal translocations occurred between genomes A_1_, A_2_, and B_1_, while genome D remained relatively stable [16]. Numerous recent reports also show that translocations occurred between chromosomes in the At-subgenome of the tetraploids [17], while no large structural variation was found in the Dt-subgenome. Therefore, we only selected SSR primers from the Dt -subgenome of the tetraploid cotton linkage map to screen putative *G. anomalum* chromosome-specific SSR primer pairs. Of the 1402 pairs of primers we selected, 1072 amplified distinct fragments in *G. hirsutum* and *G. anomalum*, including 272 dominant markers of *G. hirsutum*, 194 dominant markers of *G. anomalum* and 452 codominant markers, while 154 pairs produced no amplified polymorphic bands and another 330 pairs produced vague bands, which were excluded from further study. Then, based on the tetraploid cotton linkage map constructed by our institute [17], the above 452 codominant markers were located, and of these, 170 well-amplified and evenly distributed codominant markers within an interval of 10 cM were finally selected for use in genotyping the entire BC_1_F_1_ and BC_2_F_1_ population. The 170 codominant markers were distributed on the Dt-subgenome chromosomes, ranging from 10 to 18 markers per chromosome, with coverage of 80.9–100.0 % and a density of 6.7–15.0 cM of each chromosome (Table 2; Fig. 2). The *G. anomalum*-specific SSR markers could be used to track and identify the alien chromosomes from *G. anomalum* in *G. hirsutum*.

**Table 2:** SSR primers used for screening *G. anomalum* chromosomes in the alien addition lines

Chromosome	1B^a^	2B^a^	3B^a^	4B^a^	5B^a^	6B^a^	7B^a^	8B^a^	9B^a^	10B^a^	11B^a^	12B^a^	13B^a^
	NAU7675-120	NAU1847-200	NAU2836-230	NAU6966-200	NAU3095-260	NAU3677-160	NAU8250-220	NAU0104-230	NAU3100-170	NAU7772-160	NAU8254-160	NAU3084-250	NAU6582-550
	NAU3347-250	NAU3733-200	NAU0093-130	NAU0210-200	NAU2503-250	NAU2679-150	NAU7974-150	NAU8183-160	NAU1886-150	NAU2543-190	NAU7698-160	NAU0206-100	NAU6426-370
	NAU7914-160	NAU0645-130	NAU5675-180	NAU0012-230	NAU3183-230	NAU1454-200	NAU2556-250	NAU0738-230	NAU3888-220	NAU3917-180	NAU3731-300	NAU5397-160	NAU3011-220
	NAU3714-190	NAU8013-220	NAU0354-180	NAU0569-160	NAU0144-250	NAU1987-160	NAU2974-150	NAU2876-200	NAU6701-200	NAU4071-220	NAU0133-120	NAU7007-150	NAU7727-250
	NAU0072-180	NAU5490-280	NAU0200-410	NAU3508-200	NAU6205-160	NAU2397-270	NAU0300-120	NAU5130-320	NAU0148-170	NAU7900-150	NAU0646-140	NAU3905-150	NAU3948-250
	NAU3337-320	NAU5421-210	NAU3875-210	NAU0146-180	NAU4055-170	NAU6347-170	NAU4017-220	NAU7616-150	NAU6848-150	NAU3531-210	NAU7140-150	NAU2715-200	NAU0039-110
	NAU6624-220	NAU1778-100	NAU0088-140	NAU0033-150	NAU0378-180	NAU0783-180	NAU0121-200	NAU0583-300	NAU2753-250	NAU3665-220	NAU5418-160	NAU7838-150	NAU8306-130
	NAU0107-110	NAU6474-300	NAU3700-180	NAU7579-140	NAU6406-200	NAU0356-170	NAU3594-110	NAU5335-150	NAU0075-130	NAU0922-200	NAU6999-420	NAU8230-170	NAU2443-140
	NAU7670-150	NAU7809-200	NAU2908-200	NAU7290-230	NAU5486-200	NAU4682-150	NAU4956-280	NAU6389-270	NAU7815-250	NAU3137-300	NAU5212-200	NAU7719-200	NAU7738-160
	NAU1495-170	NAU3598-200	NAU8203-230	NAU7946-150	NAU2944-180	NAU2714-170	NAU2820-200	NAU0435-180	NAU6984-200	NAU4881-240	NAU2361-250	NAU7824-190	NAU6738-130
	NAU6095-170	NAU3820-110	NAU3292-270	NAU6993-150	NAU0123-120		NAU7686-180	NAU0069-160	NAU7743-130	NAU8079-200	NAU3373-220	NAU1274-210	NAU4871-150
		NAU1702-180	NAU5111-230		NAU6830-150		NAU2597-180	NAU3904-190	NAU0799-210	NAU0142-500	NAU2602-270	NAU8006-160	NAU3447-110
			NAU0805-190		NAU7747-160		NAU7692-150	NAU0298-130	NAU8120-320	NAU7983-170	NAU6809-160		
					NAU2655-170				NAU0245-110		NAU6315-180		
					NAU7015-150				NAU0864-240		NAU6267-180		
					NAU3826-420				NAU4477-250		NAU6520-200		
					NAU3609-250								
					NAU3656-210								
Total	11	12	13	11	18	10	13	13	16	13	16	12	12
Position	8.20-120.01	0.00-107.44	3.23-126.25	0.00-113.51	10.90-189.98	13.32-121.59	2.85-121.07	0.00-149.89	0.00-148.34	15.49-111.16	12.11-156.15	0.00-108.09	7.24-106.43
GDC (cM/)^a^	111.81	107.44	123.02	113.51	179.08	108.27	118.22	149.89	148.34	95.67	144.04	108.09	99.19
Mean density^b^	10.16	8.95	9.46	10.32	9.95	10.83	9.09	11.53	9.27	7.36	9.00	9.01	8.27
PCC (%)^c^	88.93	95.88	97.44	88.94	94.27	78.54	91.35	90.33	98.14	82.84	79.51	80.86	84.64

Note: ^a^GDC genetic distance coverage (cM); ^b^Genetic distance (cM) between two adjacent markers on a chromosome; ^c^Percentage of chromosome covered by markers (%)

### Identity of alien chromosomes from *G. anomalum* as discriminated by SSR analysis

**Fig. 3:**
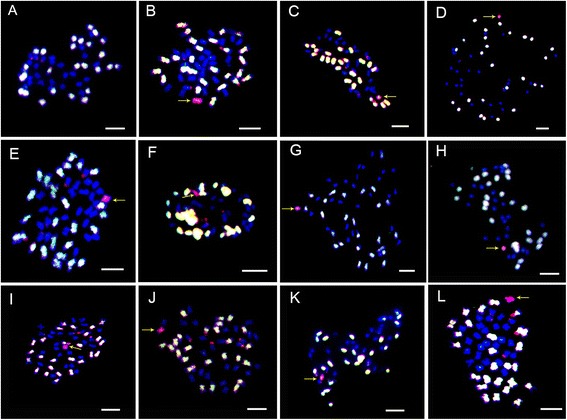
Genomic in situ hybridization of the putative monosomic alien chromosomes of *G. anomalum* in the *G. hirsutum* background using *G. herbaceum* and *G. anomalum* probes. **a** mitotic chromosome spread of the 52 chromosomes of *G. hirsutum*, showing 26 chromosomes each of the At- (white) and Dt- (blue) subgenomes. **b**-**l** mitotic chromosome spread showing the 52 *G. hirsutum* (white and blue) chromosomes and different individual chromosomes from *G. anomalum* (red), corresponding to 2B^a^ to 4G^a^ (**b**, **c** and **d**) and 6G^a^ to 13G^a^ (**e**, **f**, **g**, **h**, **i**, **j**, **k** and **l**), respectively. Scale bar = 5μm

**Fig. 4:**
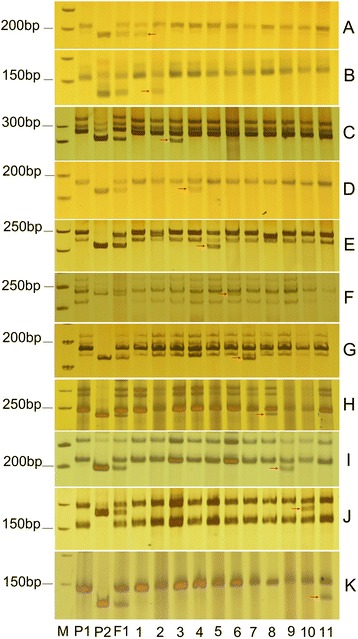
A set of *G. anomalum*-specific SSR markers were used to identify individual alien chromosomes of *G. anomalum* in *G. hirsutum*. **a**-**k** the *G. anomalum*-specific amplicons were obtained using 11 individual chromosome-specific primer pairs for markers; NAU5421, BNL2443, NAU7579, NAU3677, dPL0492, BNL2597, BNL3383, NAU4881, NAU9520, dPL0379, and dPL0864. The chromosomes correspond to D_2_ to D_4_ and D_6_ to D_13_ in cultivated tetraploid cotton. **P1**, *G. hirsutum*; **P2**, *G. anomalum*; **F1**, the hexaploid of *G. hirsutum* and *G. anomalum*; **1**-**11** show that each of these plants possesses a single different individual chromosome from *G. anomalum*, corresponding to 2B^a^ to 4B^a^, and 6B^a^ to 13B^a^. **M**, molecular size marker (50 bp ladder). Arrows (*red*) indicate chromosome-specific markers for *G. anomalum*

One hundred seventy *G. hirsutum-* and *G. anomalum*-specific codominant markers distributed on 13 Dt-subgenome chromosomes of the tetraploids were used to identify the alien chromosomes in 108 MAALs and multiple alien addition lines. The results demonstrated that 34 (31.48 %) MAAL individuals were MAAL-10B^a^ (the largest group), followed by 17 (15.74 %) MAAL-4B^a^, 16 (14.81 %) MAAL-6B^a^, 11 (10.19 %) MAAL-13B^a^, 10 (9.26 %) MAAL-2B^a^, 7 (6.48 %) MAAL-12B^a^, 3 (2.78 %) MAAL-7B^a^, 2 (1.85 %) MAAL-11B^a^, 1 (0.93 %) MAAL-3B^a^, and 1 (0.93 %) MAAL-9B^a^ (Figs. 3 and 4; Table 1). Two MAALs were not found, MAAL-1B^a^ and MAAL-5B^a^; therefore Chrs. 1B^a^ and 5B^a^ were not isolated and remained as multiple addition lines.

During the development of MAALs, Chr. 10B^a^ appeared most frequently, with an incidence of 16.87 %, followed by 15.89 % for 4B^a^, 12.47 % for 6B^a^, and 9.29 % for 12B^a^. Chrs. 5B^a^ and 9B^a^ showed very low incidences of 2.69 % and 2.44 %(Table 1).

### Morphological traits of MAALs

**Fig. 5:**
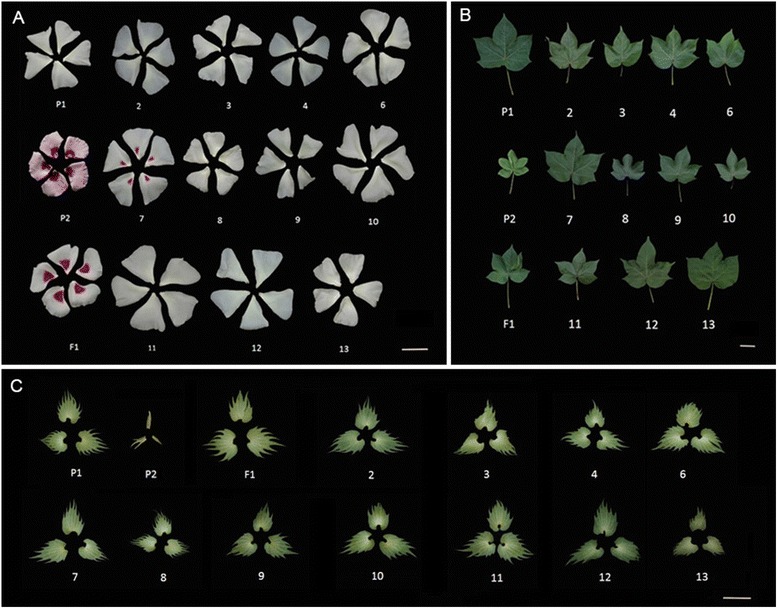
Flower and leaf traits for MAALs of *G. anomalum* individual chromosomes in *G. hirsutum*. Flower-related traits were photoed on the flowering day (0 day post anthesis, 0 DPA). **a** (petal), **b** (top third leaf) and (**c**) (bract); P1, *G. hirsutum*. P2, *G. anomalum*. F1, the hexaploid of *G. hirsutum* and *G. anomalum*. 2–4 and 6–13 are plants that carried a single different individual chromosome from *G. anomalum*, corresponding to 2B^a^, 3B^a^, 4B^a^, 6B^a^, 7B^a^, 8B^a^, 9B^a^, 10B^a^, 11B^a^, 12B^a^ and 13B^a^. Scale bar = 50 mm

**Fig. 6:**
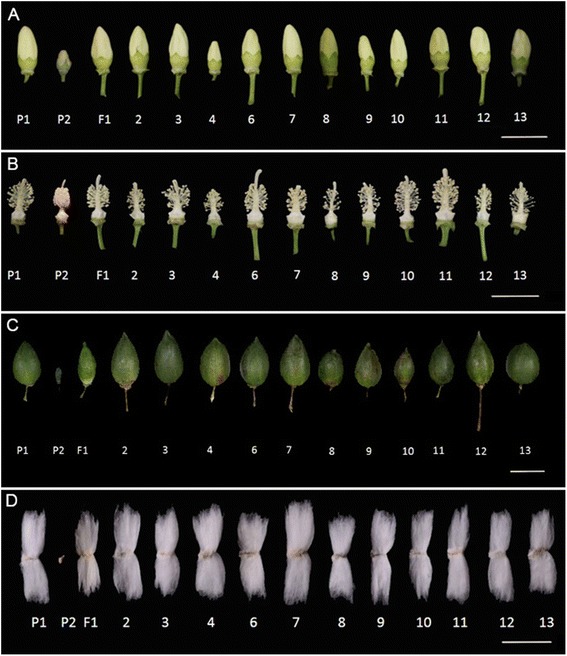
Flower, boll and fiber traits of MAALs of *G. anomalum* individual chromosomes in *G. hirsutum.* Squares, pistils and bolls were photoed at -1 DPA, 0 DPA and 35 DPA, respectively. **a** (square), **b** (pistil), **c** (boll) and **d** (fiber); P_1_, *G. hirsutum*. P_2_, *G. anomalum*. F_1_, the hexaploid of *G. hirsutum* and *G. anomalum*. 2–4 and 6–13 are plants that carried a single individual chromosome from *G. anomalum*, corresponding to 2B^a^, 3B^a^, 4B^a^, 6B^a^, 7B^a^, 8B^a^, 9B^a^, 10B^a^, 11B^a^, 12B^a^ and 13B^a^. Scale bar = 50 mm

Morphological data were gathered during the cotton growing stage. The results shown in Tables 3, 4 and 5 indicate that the eleven MAALs differed from one another and also differed from their parents in terms of their morphological traits, such as plant type, leaf shape, size of flower and boll (Figs. 5 and 6; Tables 3, 4 and 5). Most of these MAALs grew slower than the recipient, TM-1. We found that MAAL-8B^a^ leaves had a very dark green color. We also observed that MAAL-7B^a^, MAAL-12B^a^ and MAAL-13B^a^ had relatively bigger leaves, while MAAL-8B^a^, MAAL-9B^a^ and MAAL-10B^a^ had relatively smaller leaves than the other lines (Fig. 5b). In addition, MAAL-6B^a^, MAAL-10B^a^, MAAL-11B^a^ and MAAL-12B^a^ had relatively larger flowers than the others. Only MAAL-7B^a^ showed petal spots and MAAL-6B^a^ had very light brown fibers, indicating that genes for petal spots and light brown fibers are located on chromosomes 7B^a^ and 6B^a^ (Figs. 5a and 6d), respectively. MAAL-2B^a^ and MAAL-12B^a^ had relatively longer bolls and MAAL-7B^a^ had the widest boll diameter, while MAAL-8B^a^ had the shortest bolls and MAAL-10B^a^ had the smallest boll diameter (Fig. 6c). MAAL-6B^a^, MAAL-7B^a^ and MAAL-9B^a^ had a relatively larger boll weight, while MAAL-8B^a^, MAAL-10B^a^ and MAAL-11B^a^ had a relatively smaller boll weight than the others (Table 4). We found that MAAL-7B^a^ had longer fibers than the others (Fig.6d)

**Table 3:** Morphological characteristics of the eleven MAALs

Characters	TM-1	*G. anomalum*	Hexaploid F_1_	2B^a^	3B^a^	4B^a^	6B^a^	7B^a^	8B^a^	9B^a^	10B^a^	11B^a^	12B^a^	13B^a^
Petal color	Creamy	Mauve	Creamy	Creamy	Creamy	Creamy	Creamy	Creamy	Creamy	Creamy	Creamy	Creamy	Creamy	Creamy
Petal spot	Absent	Big dark red	Big dark red	Absent	Absent	Absent	Absent	light red	Absent	Absent	Absent	Absent	Absent	Absent
Petal length (cm)	4.04 ± 0.13	3.77 ± 0.49	4.75 ± 0.13	4.14 ± 0.32	4.19 ± 0.29	4.1 ± 0.32	4.37 ± 0.38	3.92 ± 0.31	3.57 ± 0.52	3.78 ± 0.51	4.49 ± 0.44	4.84 ± 0.41	4.53 ± 0.48	3.68 ± 0.21
Petal width (cm)	4.43 ± 0.20	4.37 ± 0.57	5.28 ± 0.28	4.32 ± 0.37	4.13 ± 0.22	4.01 ± 0.39	4.67 ± 0.52	4.24 ± 0.45	3.59 ± 0.66	3.76 ± 0.21	4.42 ± 0.44	5.39 ± 0.68	4.77 ± 0.58	3.53 ± 0.54
Another number	104 ± 4.97	69.33 ± 8.50	112.25 ± 10.69	96.36 ± 5.00	85.33 ± 8.08	92.50 ± 9.98	96.19 ± 12.58	68.44 ± 12.28	67.22 ± 9.39	97.40 ± 10.88	108.27 ± 9.21	109.83 ± 12.30	105.91 ± 12.24	92.09 ± 8.51
Style length (cm)	2.26 ± 0.05	1.70 ± 0.10	2.55 ± 0.17	2.19 ± 0.21	2.02 ± 0.06	1.76 ± 0.18	2.74 ± 0.24	1.78 ± 0.25	2.27 ± 0.20	2.25 ± 0.40	2.46 ± 0.32	2.60 ± 0.29	1.84 ± 0.17	2.10 ± 0.19
Stigma length (cm)	1.06 ± 0.09	0.43 ± 0.15	1.18 ± 0.15	1.09 ± 0.18	1.23 ± 0.20	0.81 ± 0.15	1.52 ± 0.26	0.83 ± 0.11	1.28 ± 0.20	1.05 ± 0.11	0.95 ± 0.38	1.51 ± 0.28	0.85 ± 0.12	1.11 ± 0.07
Pedicel length (cm)	1.05 ± 0.21	0.90 ± 0.10	1.88 ± 0.25	1.42 ± 0.40	1.22 ± 0.38	0.83 ± 0.15	2.52 ± 0.82	1.25 ± 0.34	0.78 ± 0.13	1.21 ± 0.26	1.01 ± 0.30	0.87 ± 0.27	2.97 ± 1.40	0.73 ± 0.12
sepal length (cm)	3.06 ± 0.05	1.95 ± 0.13	3.05 ± 0.17	3.17 ± 0.23	3.33 ± 0.26	2.99 ± 0.35	3.40 ± 0.29	2.98 ± 0.29	2.86 ± 0.10	2.88 ± 0.20	2.90 ± 0.29	3.19 ± 0.37	3.09 ± 0.38	2.90 ± 0.25
sepal width (cm)	1.10 ± 0.14	0.93 ± 0.10	1.00 ± 0.20	1.19 ± 0.39	1.27 ± 0.12	0.96 ± 0.14	1.12 ± 0.14	1.34 ± 0.29	0.83 ± 0.11	0.85 ± 0.12	0.87 ± 0.14	1.10 ± 0.14	1.19 ± 0.24	1.05 ± 0.22
Bracteole length (cm)	4.72 ± 0.50	1.52 ± 0.08	4.72 ± 0.32	5.28 ± 0.45	4.97 ± 0.28	4.19 ± 0.72	4.83 ± 0.63	4.84 ± 0.66	3.76 ± 0.37	4.41 ± 0.38	4.35 ± 0.58	5.20 ± 0.47	5.18 ± 0.61	3.47 ± 0.34
Bracteole width (cm)	2.85 ± 0.24	0.47 ± 0.07	2.98 ± 0.31	3.15 ± 0.35	2.57 ± 0.23	2.75 ± 0.48	3.23 ± 0.46	2.74 ± 0.45	2.47 ± 0.36	2.84 ± 0.37	2.56 ± 0.44	3.30 ± 0.27	3.16 ± 0.42	2.53 ± 0.24
Leaf color	Green	light Green	Green	Green	Green	Green	Green	Green	Dark green	Green	Green	Green	Green	Green
leaf length (cm)	12.03 ± 1.17	4.40 ± 0.36	6.57 ± 0.38	10.58 ± 2.28	9.75 ± 2.47	9.34 ± 2.25	9.10 ± 1.96	10.19 ± 1.03	7.66 ± 1.65	7.75 ± 0.21	7.70 ± 0.98	9.33 ± 3.75	10.17 ± 1.90	9.36 ± 1.74
leaf width (cm)	11.70 ± 0.20	2.53 ± 0.21	8.40 ± 0.56	11.68 ± 2.67	10.80 ± 2.69	11.46 ± 1.57	10.72 ± 2.20*	12.53 ± 1.72	10.52 ± 2.87	8.73 ± 0.11	8.40 ± 1.29	10.90 ± 3.72	11.27 ± 1.20	12.08 ± 2.10
Petiole length (cm)	6.7 ± 1.49	7.67 ± 0.47	8.57 ± 0.90	6.51 ± 2.00	4.60 ± 1.27	6.83 ± 0.85	5.71 ± 1.73	6.55 ± 1.14	6.65 ± 2.56	8.75 ± 0.503	5.60 ± 1.27	7.03 ± 3.48	7.52 ± 0.92	9.51 ± 1.69
boll length (mm)	43.08 ± 2.06	20.08 ± 1.01	33.18 ± 1.35	43.90 ± 2.94	38.75 ± 1.03	34.52 ± 1.62	34.03 ± 1.94	36.16 ± 1.41	30.58 ± 2.84	41.78 ± 0.10	34.02 ± 1.96	38.60 ± 12.00	48.88 ± 1.94	35.05 ± 2.037
boll width (mm)	39.31 ± 1.38	10.44 ± 0.61	22.34 ± 1.72	31.70 ± 3.22	41.75 ± 1.02	39.05 ± 2.19	39.82 ± 2.10	42.25 ± 2.16	31.25 ± 2.10	31.86 ± 1.82	25.52 ± 1.89	31.70 ± 2.26	33.38 ± 2.24	40.74 ± 2.54
boll tip length (mm)	3.89 ± 0.68	3.46 ± 0.59	5.06 ± 1.57	4.44 ± 0.95	4.07 ± 0.55	3.72 ± 0.85	4.94 ± 1.93	3.18 ± 0.84	3.15 ± 1.59	2.98 ± 1.71	4.08 ± 1.17	4.17 ± 1.27	5.48 ± 1.68	2.04 ± 1.10

**Table 4:** The yield-related traits of the eleven MAALs

MAAL	Boll size (g)	Seed index (g/100)	Lint percentage (%)
2B^a^	3.15	13.05	30.27
3B^a^	3.89	14.17	34.45
4B^a^	4.19	12.87	36.86
6B^a^	5.02	14.94	32.24
7B^a^	5.01	13.74	35.95
8B^a^	2.98	10.29	36.70
9B^a^	5.49	13.13	34.14
10B^a^	2.30	9.35	29.46
11B^a^	2.41	9.38	30.35
12B^a^	4.25	14.92	28.13
13B^a^	4.44	14.91	35.30
TM-1 (CK)	5.64	14.91	28.16

**Table 5:** Summary of the unique traits of the monosomic alien addition lines

MAAL	Unique traits
2B^a^	Long leaves and long calyx teeth of bract
3B^a^	Short petiole and long Sepal
4B^a^	Short column and stigma, high lint percent
6B^a^	Long column and stigma, light brown fiber
7B^a^	Purple petal spot, large leaves, long fiber
8B^a^	Small bracts and flowers with few anthers, dark green leaves
9B^a^	High boll weight
10B^a^	Small leaves and bolls, many fruit branch and bolls
11B^a^	Large flowers and the maximum anthers
12B^a^	Long tips of cone-shape bolls and long pedicels
13B^a^	Short peduncle and fruit branch, round and big bolls

## Discussion

MAALs are powerful tools in crop breeding since they can be used to produce alien translocation and substitution lines, to study interspecific relationships, and to construct single chromosome libraries. They can also be used in gene mining, gene assignment, gene expression pattern analysis, gene function analysis, physical gene mapping, isolation of chromosome-specific probes, selective isolation and mapping of cDNA/gene of a particular chromosome. Numerous reports have shown that the development of MAALs has been successfully achieved in many crops such as wheat [18–21], rice [22] tomato [23], potato [24], cucumber [25], tobacco [26], oat [12], sugar beet [27, 28], and rapeseed [29, 30]. MAALs have played and are playing important roles in numerous types of plant genomic research. The development of MAALs in *Gossypium* began as early as the 1980s but greatly lagged behind other crops due to the large number (2n = 52) and small size of chromosomes, which led to difficulty in accurately discriminating each chromosome, therefore, little progress has been made in cotton. So far only one set of MAALs has been completed [9], and this work benefited from advances in the development of GISH and molecular markers in cotton.

However, in this study, due to the very close relationship between chromosomes of the At-subgenome in *G. hirsutum* and those in *G. anomalum* often leading to cross-hybridization in GISH, we had to first improve the GISH technique by adjusting the ratio of the two different probes used. We tried five different combinations and found that the ratio of 4:3 was more suitable than any others for the discrimination of chromosomes from *G. anomalum* and the At-subgenome of *G. hirsutum.* Therefore, using a combination of the improved GISH methodology, *G. anomalum* chromosome-specific SSR molecular markers and conventional morphological survey, eleven MAALs were isolated and characterized, and two remain to be isolated from multiple addition states by further backcrossing.

Several previous reports showed that *G. anomalum* contains the favorable characteristics of drought tolerance and resistance to diseases (cotton *Verticillium* wilt, angular leaf spot) and insect pests (springtails, aphids); and more importantly, it also possesses genes with the potential to produce high quality fibers (good fiber strength and fineness) [4] and cytoplasmic male sterility [5–7]. Our previous reports also demonstrated that using *G. anomalum* as a donor parent and *G. hirsutum* as a recipient parent, a series of introgression lines with longer, stronger and finer fibers has been developed [31]. Shen et al. [32] mapped QTLs on Chr. 7 affecting fiber length in an F_2_ population derived from *G. anomalum* introgression line 7235 crossed with TM-1. However, in this study, we investigated some agronomic traits of MAALs and observed that most MAALs had poor performances in fiber quality or fiber yield components, implying that the added alien chromosomes had negative effects on most agronomic traits (Tables 4 and 6; Fig. 6). For example, the bolls of all MAALs were lighter than those of the recipient TM-1; and the fibers of all six MAALs were shorter than TM-1 (the fiber properties of the other five MAALs were not measured due to a lack of fiber samples). The resultant phenomena may be caused by linkage drag, which means that there were very close linkages between favorable and unfavorable genes on the same chromosome, even though the fibers of some MAALs were found to be stronger than those of TM-1. Therefore, to enhance the transfer of desirable genes and eliminate undesirable genes from *G. anomalum*, it is necessary to break the linkage drags to promote chromosome recombination between *G. hirsutum* and *G. anomalum*. The development of chromosome translocation lines or introgression lines may be an alternative choice based on the MAALs. We deeply believe that these MAALs of *G. hirsutum-G. anomalum* would be a powerful tool for systematically transferring desirable genes chromosome by chromosome from G. *anomalum* into G. *hirsutum*, as well as for gene mining, gene assignment, gene function analysis, gene physical mapping, isolation of chromosome-specific probes, selective isolation and mapping of cDNAs for a particular chromosome, and genomic research.

**Table 6:** Fiber quality traits from some MAALs measured by HVI

MAAL	Fiber length (mm)	Fiber uniformity (%)	Micornaire	Fiber strength (cN/tex)	Fiber elongation rate (%)
TM-1	29.08	86.20	4.35	31.95	7.00
MAAL-2B^a^	27.99	83.80	4.66	29.60	6.70
MAAL-4B^a^	26.02	83.60	4.52	28.32	6.50
MAAL-6B^a^	25.84	82.20	5.43	30.67	6.80
MAAL-8B^a^	26.99	83.40	4.04	32.44	6.60
MAAL-10B^a^	25.94	83.10	3.35	35.67	6.70
MAAL-13B^a^	27.17	84.70	4.78	28.91	6.50

## Conclusions

From this study, we draw two conclusions. (1) The GISH technique used in our previous research has been improved by using a mixture of two probes at a ratio of 4:3 (*G. anomalum* and *G. herbaceum*) to avoid cross-hybridization caused by the very close relationship between chromosomes from *G. anomalum* and the At-subgenome of *G. hirsutum*, which can be suitable for recognizing alien chromosomes of *G. anomalum* in *G. hirsutum* background. (2) Eleven out of 13 potential MAALs were isolated, which would be used, at the chromosome level, for effectively identifying favorable genes in *G. anomalum*, allowing for more accurate and faster transfer of such genes to create introgression lines, the effect of specific alien chromosomes to be examined, homeologies with chromosomes of cultivated species to be compared, and physical maps of specific chromosomes to be constructed.

## Methods

### Plant materials

In 2012, the amphiploid (allohexaploid) (2n = 6× = 78, AADDBB genome) (previously obtained in our institue) derived from the doubled triploid hybrid of *G. hirsutum* (2n = 4× = 52, AADD genome) × *G. anomalum* (2n = 2× = 26, BB genome, obtained from Cotton Research Institute of Chinese Academy of Agricultural Sciences) was backcrossed as a maternal parent with *G. hirsutum* acc TM-1, the genetic standard line of upland cotton*.* In 2013, two pentaploid individuals were obtained at Pailou Experimental Station of Nanjing Agricultural University (PES/NJAU) and used as both paternal and maternal parents in the backcross with TM-1. The BC_1_ seeds obtained were planted in plastic cups with sterilized soil and incubated in the phytotron at Nanjing Agricultural University in 2014 spring at 25–28 °C and with 80% relative humidity. When they reached the fifth true leaf stage, the seedlings were transplanted into clay pots at PES/NJAU. Lastly, 38 BC_1_ individuals were identified using SSR markers and GISH and consecutively backcrossed with TM-1. The BC_2_ seeds obtained were planted in the same way in spring 2015. In the winter, all plants were moved into the greenhouse at PES for preservation.

### Scheme for developing the monosomic alien addition lines

The interspecific hexaploid was backcrossed with *Gossypium hirsutum* acc TM-1 (obtained from the Southern Plains Agricultural Research Center, USDA-ARS) to produce the pentaploid (2n = 5× = 65, AADDB genome), then the pentaploid progenies were further consecutively backcrossed with TM-1 to generate backcross progenies (BC_1_ and BC_2_). GISH was used to characterize alien chromosomes in all backcross progenies from the BC_1_ generation. When more than one alien chromosome was added from *G. anomalum*, the progenies were further backcrossed with TM-1 to produce monosomic alien addition lines. If only one alien chromosome was added to the background of Upland cotton, the progenies were further examined using chromosome-specific SSR markers of *G. anomalum* to determine the identity of the added chromosome.

### *G. anomalum*, TM-1, BC_1_, and BC_2_ chromosome preparation

Cotton seeds were cultivated in an incubator at 29 °C and their root tips were cut off when they grew to 3 cm long (seedling plant). The tips were immersed in 25 μg/ml cycloheximide at room temperature for 2 h to accumulate metaphase cells and then transferred to Carnoy I fixative containing 95% ethanol and acetic acid (3:1, v/v) for at least 2 h, digested in double enzymolysis liquid (4 % cellulose: 1 % pectinase = 1:2) at 37 °C for 45 min, and squashed in a drop of 45 % acetic acid. Finally, slides containing at least 20 well-spread somatic chromosomes at mitotic metaphase were prepared and stored at -70 °C overnight.

### Genomic in situ hybridization (GISH)

Due to the very close relationships that exist between chromosomes of the B genome in *G. anomalum* and those of the At subgenome in *G. hirsutum*, two probes were employed here to avoid cross-hybridization between these chromosomes. Genomic DNA extracted from *G. anomalum* and *G. herbaceum* (2n = 2× = 26, AA genome) were labeled with digoxigenin-11-dUTP and Bio-16-dUTP (Roche Diagnostics, Mannheim, Germany) by nick translation, respectively. The probe fragment size was between 200-500 bp. Fluorescence in situ hybridization was carried out as described by [33] and [9] with some modifications. The mixing ratio of DNA probes from *G. anomalum* and *G. herbaceum* were adjusted to five different ratios, 2:1, 4:3, 1:1, 2:3, and 1:2, to determine the optimal ratio for discrimination of chromosomes from *G. anomalum* and the At-subgenome of *G. hirsutum*.

### DNA extraction and *G. anomalum*-specific primer screening

Genomic DNA was extracted from young leaves of the two parents, *G. anomalum* and *G. hirsutum* acc. TM-1, the interspecific hexaploid, the pentaploid, and the BC_1_ and BC_2_ individuals using the method described by [34] with some modifications. A total of 2,168 pairs of SSR primers were selected from the high density genetic linkage map of Sea island and Upland cotton constructed in our institute [17] and employed to screen *G. anomalum*-specific primers. PCR reactions were performed and their amplified products were separated by PAGE, as described by [35, 36]. The *G. anomalum*-specific marker primers obtained were further used to characterize each chromosome from *G. anomalum*.

### MAAL nomenclature

Thirteen *G. hirsutum-G. anomalum* MAALs were named MAAL-1B^a^ to MAAL-13B^a^, according to the method described by [9], in which B represents the B genome of *G. anomalum* and ‘a’ refers to the initial letter of *anomalum*. The chromosome numbers 1 to 13 in the B genome of *G. anomalum* correspond to the homoeologous chromosomes in the Dt-subgenome of tetraploid cotton.

### Investigation of agronomic traits of monosomic alien addition line

At the point of transition from the vegetative to the reproductive stage, the shape and size of fully expanded leaves from the same position in TM-1, *G. anomalum*, hexaploid and MAAL plants were investigated. Floral morphological traits from these MAALs were investigated in the flowering period. The size of cotton bolls at 35 days post-anthesis was also measured by vernier caliper. Finally, the hundred-seed weight, ginning outturn and single boll weight of the matured bolls were investigated. All the data were analyzed using the SPSS software version 18.0.

## Additional file

Additional file 1: Table S1. Incidence of alien chromosomes in the BC_1_ *G. hirsutum* × *G. anomalum* generations. (DOC 33 kb)

## Abbreviations

GISH: Genomic in situ hybridization
MAAL: Monosomic alien addition line
SSR: Simple sequence repeat
