# Supplementary material for: Characterization of eleven monosomic alien addition lines added from Gossypium anomalum to Gossypium hirsutum using improved GISH and SSR markers
Source: BMC Plant Biol. 2016 Oct 7;16:218. doi: 10.1186/s12870-016-0913-2 (PMC5055718; doi:10.1186/s12870-016-0913-2)
Supplement: Additional file 1: Table S1. — Incidence of alien chromosomes in the BC1 G. hirsutum × G. anomalum generations. (DOC 33 kb) [file 12870_2016_913_MOESM1_ESM.doc]

**Additional file 1: Table S1.** Incidence of alien chromosomes in the BC_1_ *G. hirsutum* × *G. anomalum* generations

| Chromosome number | individual | Proportion (%) |
| --- | --- | --- |
| 52 | 1 | 2.63 |
| 52+1 | 2 | 5.26 |
| 52+2 | 4 | 10.53 |
| 52+3 | 7 | 18.42 |
| 52+4 | 5 | 13.16 |
| 52+5 | 3 | 7.89 |
| 52+6 | 8 | 21.05 |
| 52+7 | 2 | 5.26 |
| 52+8 | 2 | 5.26 |
| 52+9 | 2 | 5.26 |
| 52+13 | 2 | 5.26 |
| Total | 38 | 100.00 |
